# Cross-modality supervised image restoration enables nanoscale tracking of synaptic plasticity in living mice

**DOI:** 10.1038/s41592-023-01871-6

**Published:** 2023-05-11

**Authors:** Yu Kang T. Xu, Austin R. Graves, Gabrielle I. Coste, Richard L. Huganir, Dwight E. Bergles, Adam S. Charles, Jeremias Sulam

**Affiliations:** 1 Department of Neuroscience, Johns Hopkins University School of Medicine, Baltimore, MD USA; 2 Kavli Neuroscience Discovery Institute, Johns Hopkins University, Baltimore, MD USA; 3 Department of Biomedical Engineering, Johns Hopkins University School of Engineering, Baltimore, MD USA; 4 Center for Imaging Science, Johns Hopkins University, Baltimore, MD USA

**Keywords:** Synaptic plasticity, Optical imaging, Machine learning, Image processing, Mouse

## Abstract

Learning is thought to involve changes in glutamate receptors at synapses, submicron structures that mediate communication between neurons in the central nervous system. Due to their small size and high density, synapses are difficult to resolve in vivo, limiting our ability to directly relate receptor dynamics to animal behavior. Here we developed a combination of computational and biological methods to overcome these challenges. First, we trained a deep-learning image-restoration algorithm that combines the advantages of ex vivo super-resolution and in vivo imaging modalities to overcome limitations specific to each optical system. When applied to in vivo images from transgenic mice expressing fluorescently labeled glutamate receptors, this restoration algorithm super-resolved synapses, enabling the tracking of behavior-associated synaptic plasticity with high spatial resolution. This method demonstrates the capabilities of image enhancement to learn from ex vivo data and imaging techniques to improve in vivo imaging resolution.

## Abstract (web summary)

XTC is a supervised deep-learning-based image-restoration approach that is trained with images from different modalities and applied to an in vivo modality with no ground truth. XTC’s capabilities are demonstrated in synapse tracking in the mouse brain.

## Main

Synaptic plasticity is a widely studied model of behavioral learning and memory encoding^1–3^ that directly links molecular changes at synapses to changes in the flow of information through neural circuits. Synaptic potentiation is observed during learning, whereas synaptic deficiency is observed in many neurological diseases^4–6^; however, observing how changes in synaptic strength manifest during learning in behaving animals is difficult due to the lack of biological tools for visualizing the strength of synapses and the limited resolution of fluorescence microscopy in vivo. Overcoming these technical constraints is vital to understanding how learning is encoded in real time among billions of synapses in the brain.

Genetically encoded fluorescent tags enable direct visualization of protein expression in vivo. For instance, by fusing super-ecliptic pHluorin (SEP)—a pH-sensitive variant^7^ of green fluorescent protein (GFP)—to the extracellular domain of AMPA-type glutamate receptors (AMPARs), it is possible to directly visualize the insertion and recycling of these crucial proteins at the synaptic membrane. Functional SEP-tagged AMPARs, inserted at the cell surface, fluoresce in the neutral pH of the extracellular space, whereas internalized SEP-tagged AMPARs have their fluorescence quenched by the low internal pH of trafficking vesicles. As AMPARs mediate excitatory neurotransmission, the fluorescence intensity of SEP-tagged AMPARs can be used as a measure of synaptic strength. This transgenic approach has recently been employed to track changes in synaptic strength in living animals^8–12^.

While SEP-labeling theoretically enables the visualization of all surface AMPARs in living mice, achieving sufficient resolution to reliably track SEP-tagged synapses in vivo presents a substantial challenge, as synapses are submicron-diameter structures that are present at high density^13^. Moreover, as synapses vary in AMPAR content, endogenous SEP fluorescence is dim at some synapses. Thus, to image SEP signals in vivo, a balance must be achieved between imaging resolution, depth, speed and laser power. While two-photon (2p) microscopy is state of the art for in vivo imaging^14,15^, the maximum resolution of 2p imaging falls behind that of single-photon (1p) microscopy in vitro^14^. Axial resolution is especially impaired in 2p microscopy, as there is no pinhole for optical sectioning and the long working distance required for in vivo imaging means that high numerical aperture (NA) objectives cannot be used. Moreover, adapting in vitro super-resolution microscopy elements, such as Airyscan detectors, to in vivo imaging is also difficult, as depth-dependent light scattering, movement artifacts and tissue swelling, force compromises between acquisition resolution, size, depth and photobleaching. As such, current methodologies only allow for live imaging of molecular synaptic changes in ex vivo brain slice preparations.

To overcome these limitations, we developed a machine-learning system to combine the advantages of both in vitro and in vivo imaging modalities. Convolutional neural networks (CNNs) serve as one promising avenue to selectively balance the benefits of different imaging modalities^16–23^. Unlike traditional restoration algorithms^24,25^, deep-learning models, such as content-aware image restoration (CARE^17^), learn application-specific information from training data, thereby adapting to the high complexity of signals from living animals; however, the necessity to learn data statistics from paired training data (high- and low-resolution images of the same tissue) is difficult in scenarios where optimal-resolution data are lacking, such as identifying synapses in vivo and tracking their plasticity during behavior^26,27^. We circumvented this limitation by developing an approach that leverages paired data across different imaging modalities to train a restoration algorithm, which we termed cross-trained CARE (XTC).

In this Article, we apply XTC to restore low-resolution in vivo 2p data acquired from a transgenic mouse line that we generated, SEP–GluA2, allowing us to visualize the strength of individual excitatory synapses over weeks. XTC outperformed existing state-of-the-art image-denoising algorithms^28–31^, facilitating reliable, longitudinal synapse tracking in regions of high synapse density. By combining the advantages of multiple imaging modalities, deep-learning and transgenic labeling, this platform provides a general means to study objects that are near the diffraction limit in vivo, while also specifically enabling researchers to explore the role of synaptic plasticity in learning and memory with high resolution.

## Results

### Transgenic SEP label visualizes individual synapses in vivo

**Fig. 1:**
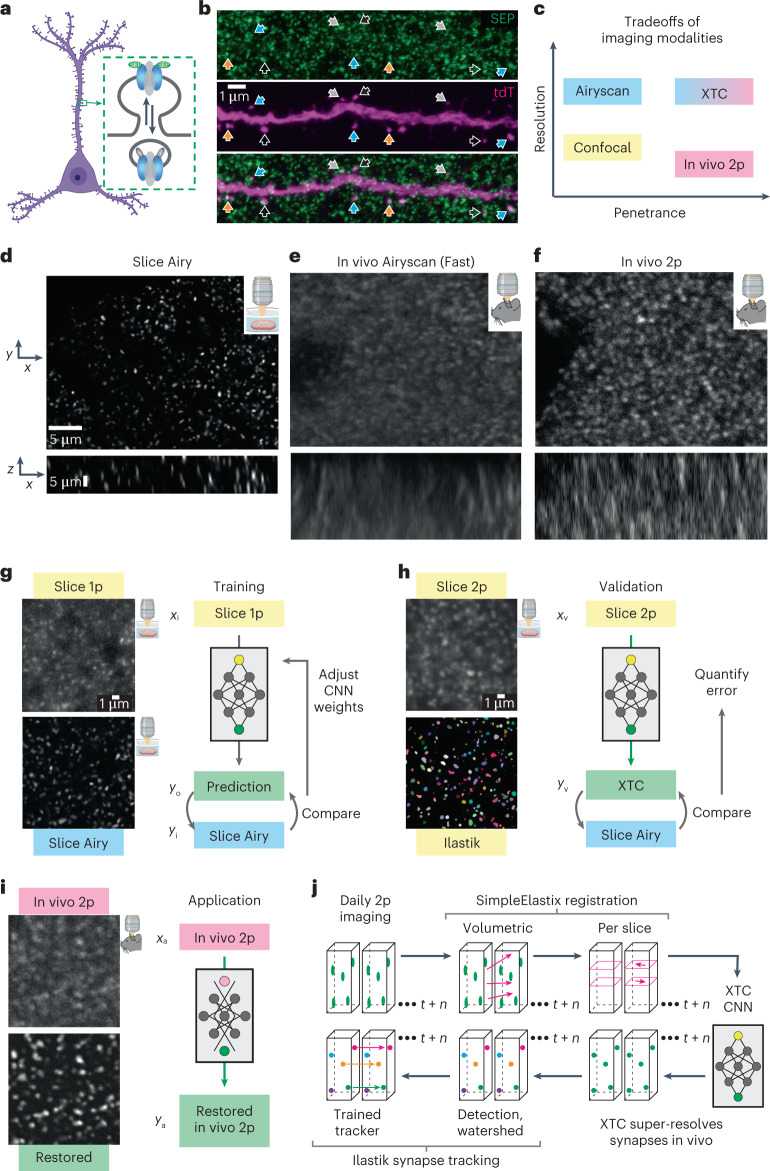
Resolving AMPAR clusters at individual excitatory synapses in vivo. **a**, CRISPR-based transgenic labeling of the GluA2 AMPA receptor subunit with a pH-dependent fluorescent tag (SEP) enables in vivo visualization of endogenous GluA2-containing synapses. **b**, Single high-resolution imaging plane from fixed-slice tissue with endogenous fluorescence, acquired using Airyscan detectors. Magenta, tdT; green, SEP–GluA2. Arrows mark examples of SEP–GluA2/spine overlap. Colored arrows show the same synapse across image channels. Data are representative of three SEP–GluA2 mice examined over one independent experiment. **c**, Tradeoffs of different imaging modalities. **d**–**f**, Example *xy* slice (top) and *xz* slice (bottom) of different imaging modalities. Scale bar, 5 µm in *xy* and *z*. Data are representative of three SEP–GluA2 mice, each imaged with all three microscopy modalities in three independent trials. **g**–**i**, Diagrams of training, validation and application workflow. Representative images of single *xy* plane of each color-coded imaging modality (left). Workflow of training, validation or application (right). Insets are representative images of tissue from six SEP–GluA2 mice, examined over three independent experiments. CNN was trained using 1p confocal images from acute slices of SEP–GluA2 tissue (*x*_i_). CNN output (*y*_o_) was compared to ground truth (high-resolution Airyscan imaging of the same tissue, *y*_i_) to improve network performance (**g**). Network output was validated by comparing to ground truth and annotations by expert humans, enabling quantification of error rates (**h**). Trained restoration CNN was applied to in vivo 2p images, restoring optimal ‘Airyscan-like’ resolution to in vivo imaging volumes (**i**). *x*_v_, slice 2p data; *y*_v_, XTC Restored slice 2p data; *x*_a_, raw in vivo 2p data; *y*_a_, XTC Restored in vivo 2p data. **j**, Pipeline for longitudinal tracking of fluorescently labeled SEP–GluA2 synapses in vivo. Daily imaging volumes were aligned using pairwise affine registration, followed by slice-by-slice pairwise affine registration to compensate for depth-dependent local tissue shift. Registered volumes were restored with XTC. Individual synapses were segmented with an ilastik-trained random forest model, followed by watershed to separate adjacent objects. Finally, a tracker trained through structured learning was used to longitudinally track synapses. *t* indicates current timepoint; *n* indicates number of subsequent timepoints.

To visualize synapse dynamics in the intact brain of living animals, we used CRISPR-Cas9 DNA editing to create a transgenic mouse line with fluorescently labeled endogenous AMPARs. In homozygous SEP–GluA2 mice, each GluA2 AMPAR subunit is fused with SEP. By coupling SEP to the extracellular N terminus of the receptor, only GluA2-containing AMPARs at the cell surface are highly fluorescent (Fig. 1a). Notably, as the overwhelming majority of AMPARs at excitatory synapses in the cerebral cortex contain GluA2^32^, this transgenic line enables visualization of nearly all excitatory synapses in this region. To assess the spatial association of SEP fluorescence with physical synaptic sites, we used super-resolution confocal microscopy (Airyscan) to image brain slices from SEP–GluA2 mice in which layer 2/3 pyramidal neurons were induced to express tdTomato (tdT). Dual-channel imaging revealed that SEP–GluA2 puncta colocalize with tdT fluorescent (tdT^+^) dendritic spines (Fig. 1b), suggesting normal synaptic targeting of fluorescently tagged receptors.

Optically, resolving the dense and dim endogenous SEP signal in the intact brain is challenging, particularly due to motion artifacts associated with in vivo imaging. Using cranial windows implanted over retrosplenial cortex, we tested several imaging modalities (confocal, Airyscan in vitro, Airyscan Fast in vivo^33^, 2p with galvanometric scanner and 2p with resonance scanner) (Fig. 1c–f) but were unable to reliably resolve adjacent synapses in vivo, particularly in the axial plane. While the best resolution was achieved using 2p excitation and a resonance scanner that reduced motion artifacts (Fig. 1f), none of these methods preserved the overall shape and clarity of fluorescent synapses observed using Airyscan microscopy in brain slice preparations (Fig. 1d). Thus, to improve synapse detection in 2p imaging datasets, we sought to combine the resolution of Airyscan microscopy with the speed and penetration of 2p excitation using computational image restoration (Fig. 1c).

### Cross-modality pairing enables in vivo restoration model

**Extended Data Fig. 1:**
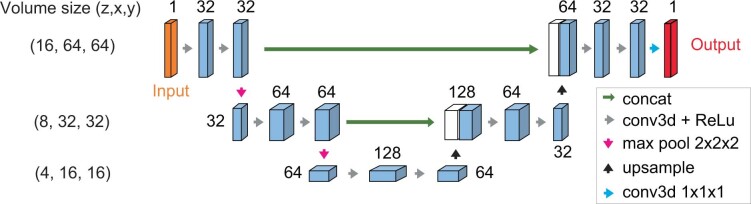
XTC network architecture for image restoration. Architecture based on 3D U-Net^34^. Single channel input volume is convolved and downsampled along the descending branch of the network. The ascending branch then upsamples the data, such that the restored output is identical in size to the input volume. Convolutional kernels 5 × 5 × 5 unless otherwise indicated.

Computational image restoration offers adaptable methods to overcome limitations associated with specific optical systems^16–23^. Using supervised training of a CNN to enhance the image quality of a suboptimal imaging modality to that of a higher resolution target dataset, researchers can, in principle, selectively balance the advantages of different imaging modalities. In the present application, the speed and penetrance of 2p microscopy, which facilitates in vivo imaging, needs to be combined with substantially higher resolution Airyscan microscopy; however, acquiring such a paired dataset is not feasible, as 2p datasets represent the upper limit of data quality in current in vivo optical applications (Fig. 1). Thus, to improve the resolution of fluorescently labeled synapses in vivo, we performed in vitro imaging of acute slices of SEP–GluA2 brains to produce a training dataset from which a restoration algorithm could learn a mapping strategy from low-resolution 1p confocal (Slice 1p) images to high-resolution Airyscan image quality (Slice Airy; Fig. 1g). We hypothesized that images from acutely prepared, living brain slice preparations, acquired immediately after dissection in physiological buffers to preserve tissue quality and pH gradients that are critical for SEP fluorescence, would be sufficiently similar to in vivo datasets, such that a restoration CNN trained using paired live-slice training data would enhance the resolution of in vivo images. Thus, we defined ground truth high-resolution data (Slice Airy) as images acquired with Airyscan super-resolution microscopy, whereas low-resolution paired data (Slice 1p) was generated using 1p excitation with an open pinhole, decreased laser power and high gain, to resemble the axial blur and high noise of in vivo 2p imaging (Fig. 1g–i). A CNN with a modified U-Net architecture^34^ was then trained using this paired high–low training data to generate a restoration model, termed XTC (Extended Data Fig. 1). After training the image-restoration algorithm, we assembled an analysis pipeline to enable synapse tracking across longitudinal imaging experiments (Fig. 1j).

### XTC restores high-resolution synaptic signals in vivo

**Fig. 2:**
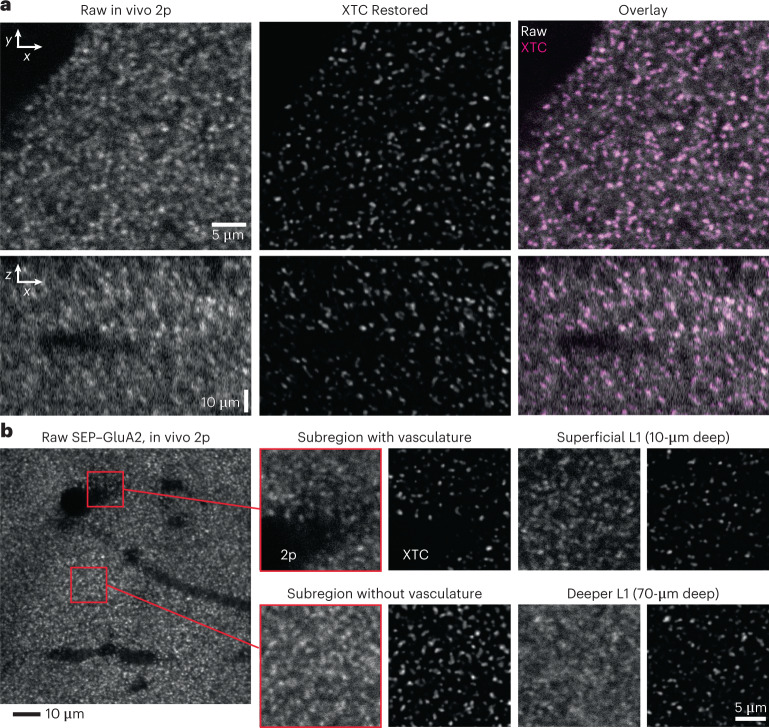
XTC super-resolves SEP synapses in vivo. **a**, Comparison of same in vivo 2p image before (left) and after XTC (middle). All images show a single axial slice. **b**, Representative slice from a single volume acquired in vivo (100 × 100 × 70 µm). Zoomed insets show XTC performance near-blood-vessel obstructions, in sparse and dense regions and deeper in the cortex. All figures show single *xy* section of volume, not maximum projections. Full volumetric comparisons are shown in Supplementary Videos 1 and 2. Data are representative of four SEP–GluA2 mice, examined over two independent experiments.

**Extended Data Fig. 2:**
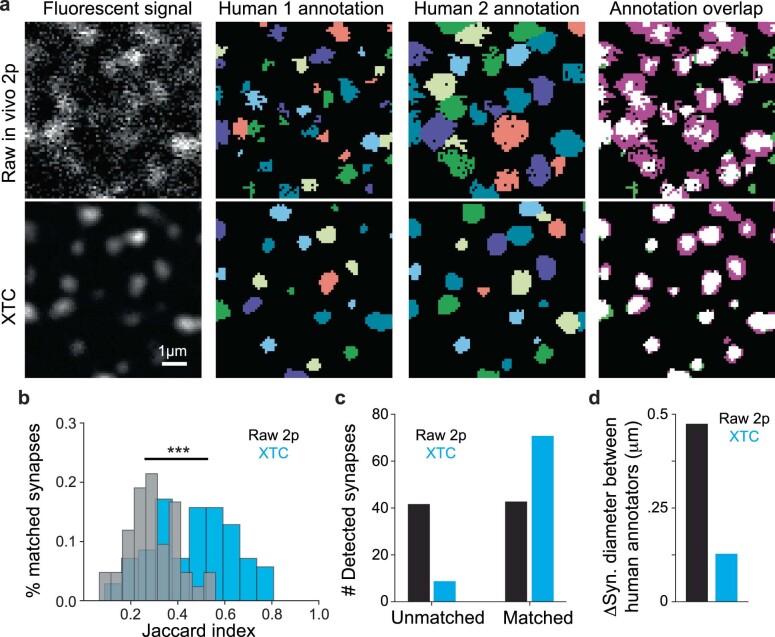
Inter-researcher variability in synapse detection is reduced with XTC restoration. (**a**) Representative slices from human annotated volume of SEP-GluA2 labeled synapses, both with and without XTC processing. Tracing was performed in ITK-SNAP^56^ using a brush tool by human expert 1 and 2 and overlaid on the far right. Data representative of synapses from one SEP-GluA2 mouse. (**b**) Distribution of segmentation similarity, as indicated by Jaccard index, between matched synapses traced by both experts. 0.29 ± 0.02 *Raw 2p* and 0.46 ± 0.02 µm *XTC* mean Jaccard index, *p* = 7.7 × 10^−8^; unpaired two-tailed t-test. (**c**) Number of unmatched and matched synapses between human annotators (defined as having at least 75% shared voxels) before and after XTC restoration. (**d**) The absolute difference in mean synapse diameter between human annotators before and after XTC restoration.

In vivo 2p images restored using the XTC model exhibited improved lateral and axial resolution (Fig. 2a and Supplementary Videos 1 and 2). Closer inspection of distinct regions with sparse synapses, dense synapses and near-blood-vessel occlusions or in volumes with depth-dependent signal loss demonstrated that the CNN adapted to regional changes in image statistics to faithfully preserve visible synapses (Fig. 2b). Moreover, when two human experts were tasked with annotating the same volume, before and after XTC restoration, XTC processing significantly improved segmentation similarity between researchers (mean ± s.e.m. Jaccard index 0.29 ± 0.02 Raw 2p and 0.46 ± 0.02 XTC; *P* = 7.7 × 10^–8^; unpaired two-tailed Student’s *t*-test; Extended Data Fig. 2b), indicating that XTC restoration facilitates reliable, reproducible analysis by reducing inter-researcher variability in synapse detection.

**Fig. 3:**
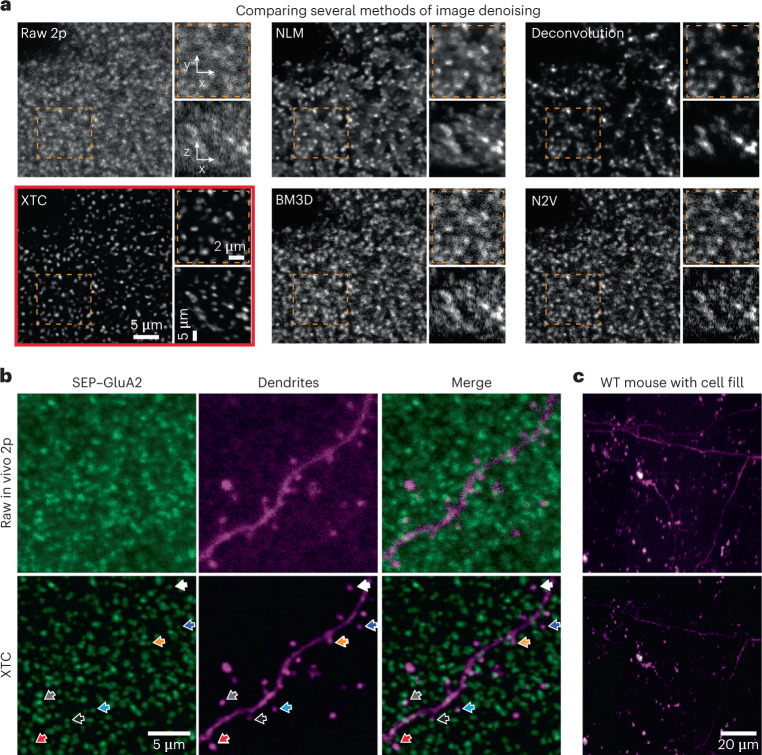
XTC outperforms existing algorithms and generalizes to other signals in vivo. **a**, Comparison of XTC restoration to four algorithms (NLM, Deconvolution, BM3D and Noise2Void). Identical ‘Raw 2p’ volume provided to all algorithms. Inset crop from orange boxes shown on right of each comparison, with *xy* and *xz* projections shown at the top and bottom, respectively. All figures show a single section of volume, not maximum projections. Full volumetric comparisons are shown in Supplementary Video 3. Data are representative of larger volume from one SEP–GluA2 mouse, examined over four independent experiments. **b**, Application of SEP-trained XTC model to alternative in vivo fluorescent signals. Super-resolved dendrite and associated SEP–GluA2 spines super-resolved with XTC (bottom, colored arrows show same synapse across image channels). Data are representative of four SEP–GluA2 mice, examined over one independent experiment. **c**, Application of SEP-trained XTC model to wild-type (WT) mice with no SEP–GluA2 synapses (tdTomato cell fill magenta). Identical beam paths, laser power, PMT gain and display settings were used in **b** and **c**.

To compare the performance of XTC to existing denoising methods, we employed four algorithms of varying complexity: (1) non-local means (NLM) represents a simple standard image-denoising baseline^28^; (2) Deconvolution^29^ and (3) block-matching and three-dimensional filtering (BM3D)^35^ represent traditional denoising algorithms; and (4) Noise2Void (N2V) represents a modern unsupervised deep-learning approach^31,36^. When these methods were applied to restore the same in vivo 2p data, we observed that XTC achieved superior resolution (Fig. 3 and Supplementary Video 3), improving background denoising while enhancing signal quality in both lateral and axial dimensions. Alternative methods struggled to balance background subtraction with signal retention. For instance, Deconvolution was able to amplify synaptic signals, but suffered from signal loss due to excessive denoising (Fig. 3a). The superior performance of XTC demonstrates the necessity of supervised deep-learning approaches to achieve robust image denoising in vivo.

**Extended Data Fig. 3:**
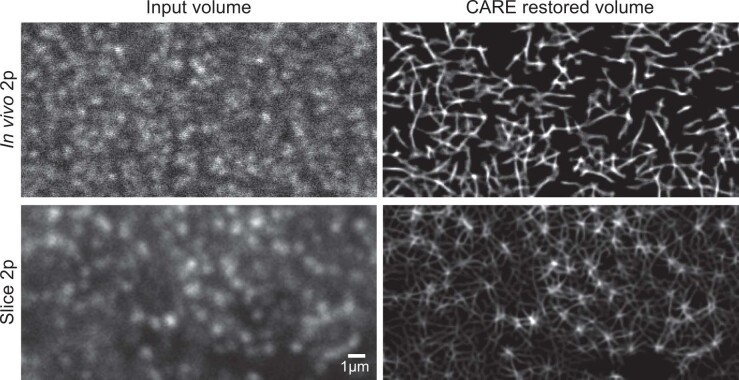
Pre-trained CARE models hallucinate when applied to synapse data. Application of pre-trained CARE restoration algorithm (trained on a microtubules dataset) to SEP-GluA2 input volume. Hallucinated microtubules visible when applied to both in vivo *2p* and *Slice 2p* data. Data are representative of larger volumes from two SEP-GluA2 mice, examined over two independent experiments.

As the functions parametrized by CNNs are complex, it is also important to empirically test for artifactual errors, such as ‘hallucinated’ false-positive synapses (Extended Data Fig. 3). Testing for false positives also demonstrates how well XTC generalizes beyond the data distribution used for training. First, we applied XTC, with no additional training, to a structural neuronal signal: viral expression of tdT. We observed that restored images faithfully retained the linear properties of dendrites and did not create hallucinated spherical ‘synapse-like’ objects (Fig. 3b). Moreover, after enhancing both tdT and SEP signals independently using XTC, SEP-labeled synapses remained colocalized with dendritic spines, showing that the spatial distribution of SEP synapses was not altered through image restoration (Fig. 3b). Finally, we also tested XTC on wild-type animals (not expressing SEP) with tdT-filled neurons and found no false positives (Fig. 3c). Together, these results indicate that XTC faithfully enhances synaptic fluorescent signals in vivo without distorting their underlying shape and visualized distribution.

### Assessment of XTC performance in vitro

Preserving the intensity and size of SEP-labeled synapses is critical to accurately assess changes in AMPAR expression, as SEP fluorescence is directly correlated with synaptic strength^8^. To assess how image restoration impacts synapse shape, intensity and spatial distribution, we generated validation data by pairing high-resolution volumes, imaged with Airyscan microscopy (Slice Airy), with low-resolution volumes, imaged with 2p excitation (Slice 2p; Fig. 1g, h) in slice tissue.

**Fig. 4:**
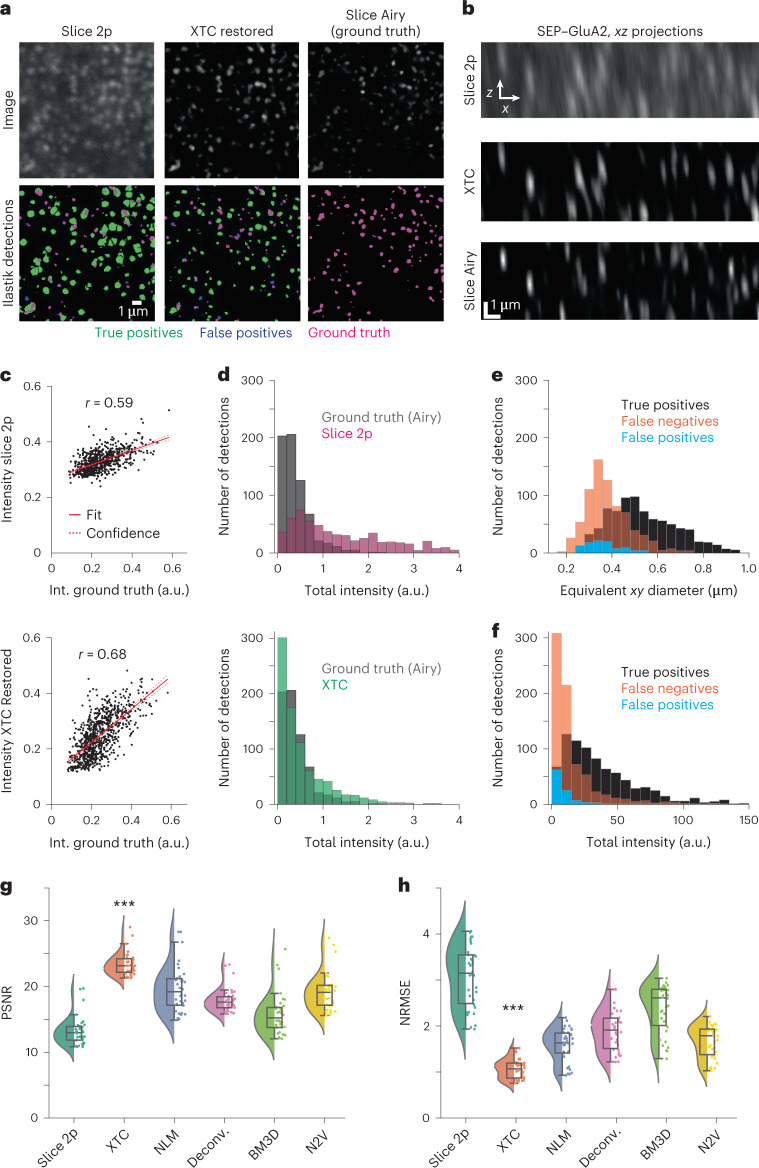
Validation of automated synapse detection following XTC. **a**, Comparison of signals throughout XTC workflow used for validation (top). Synapse detections from top row volumes using trained ilastik synapse segmentation models (bottom). True positives (green), false positives (blue) and false negatives/ground truth (magenta) are indicated. **b**, Comparison of lateral resolution before and after XTC restoration. Data are representative of larger volumes from three SEP–GluA2 mice, examined over one independent experiment. **c**, Correlation of mean fluorescence intensity of matched segmented synapses from Slice 2p or XTC Restored volumes to segmented synapses from the ground-truth volume. a.u., arbitrary units. **d**, The distribution of total intensity (sum of total voxel intensity within a segmented synapse) for individual synapses in XTC Restored, Slice 2p and ground-truth volumes. **e**,**f**, The distribution of synapse diameter (**e**) and total intensity (**f**) for paired true-positive, false-negative and false-positive detections in XTC Restored volume relative to Slice Airy validation data. **g**,**h**, PSNR and NRMSE comparisons across techniques. All comparisons with XTC, PSNR: Slice 2p (*P* = 2.0 × 10^−34^), NLM (*P* = 2.1 × 10^−7^), Deconvolution (Deconv.) (*P* = 1.4 × 10^−13^), BM3D (*P* = 10^−22^) and N2V (*P* = 5.7 × 10^−9^); NRMSE: Slice 2p (*P* = 2.0 × 10^−50^), NLM (*P* = 10^−6^), Deconv. (*P* = 3.9 × 10^−14^), BM3D (*P* = 2.8 × 10^−28^) and N2V (*P* = 2.6 × 10^−8^); *n* = 80 images per condition, one-way analysis of variance with Bonferroni correction for multiple comparisons. ^∗∗∗^*P* < 0.001. Box-plot elements are defined as follows: center line (median); box limits (upper and lower quartiles); whiskers (1.5× interquartile range); points represent individual cropped images (points outside of whiskers are outliers). Sample outputs shown in Extended Data Fig. 6.

**Extended Data Fig. 4:**
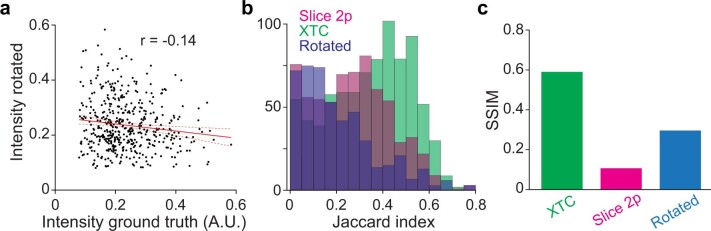
Additional comparison of synapses detected before and after XTC restoration. To serve as a semi-randomized baseline, the Slice Airy volume was rotated 90°. (**a**) Correlation of the mean intensity of matched segmented synapses between rotated and unrotated Slice Airy volumes (r = −0.14). (**b**) Optimal overlap similarity (Jaccard index) between matched segmented synapses between Slice Airy and either Slice 2p, XTC or rotated volumes. (**c**) The structural similarity index (SSIM) for full volumes, as compared between Slice Airy and either *Slice 2p*, XTC or rotated data.

**Extended Data Fig. 5:**
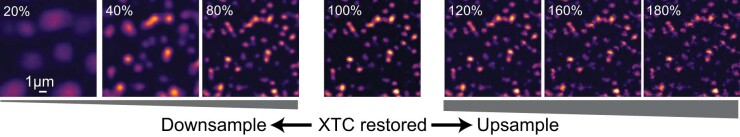
XTC performs optimally within scale of training data. Assessment of varied image XY resolution (scaling) on XTC model performance. Volumes tested between range of 20 – 180% of training data resolution. Training data was acquired between 0.063–0.095 µm/px in XY resolution. Data are representative of larger volumes from three SEP-GluA2 mice, examined over one independent experiment.

To compare individual synapses before and after XTC restoration to synapses from Slice Airy validation data, we trained random forest classifiers using ilastik^37^ to volumetrically segment individual synapses. Three ilastik models were trained, each using 30 sparse, human annotations, to detect synapses in Slice 2p, XTC Restored and Slice Airy (ground truth) volumes, respectively (Fig. 4a,b). When comparing ilastik classifiers, we concluded that XTC restoration facilitated synapse segmentation, as each pair of segmented synapses, between XTC Restored and Slice Airy images, was more similar in size, shape and intensity than each pair of segmented synapses between Slice 2p and Slice Airy images (Fig. 4). Overall, XTC image restoration preserved the correlation of mean intensity values for individual synapses relative to ground truth (Fig. 4c; *r* = 0.59, Slice 2p and *r* = 0.68, XTC Restored) and improved the distribution of total sum intensity for individual synapses to better match the total sum intensity distribution of individual synapses in Slice Airy data (Fig. 4d). Moreover, the overall shapes of XTC Restored synapses were better matched to ground truth detections, as indicated by the Jaccard overlap index^38^ (Extended Data Fig. 4). Notably, XTC performed optimally when provided with input data that were within the resolution scale of the training dataset, as expected (Extended Data Fig. 5).

**Extended Data Fig. 6:**
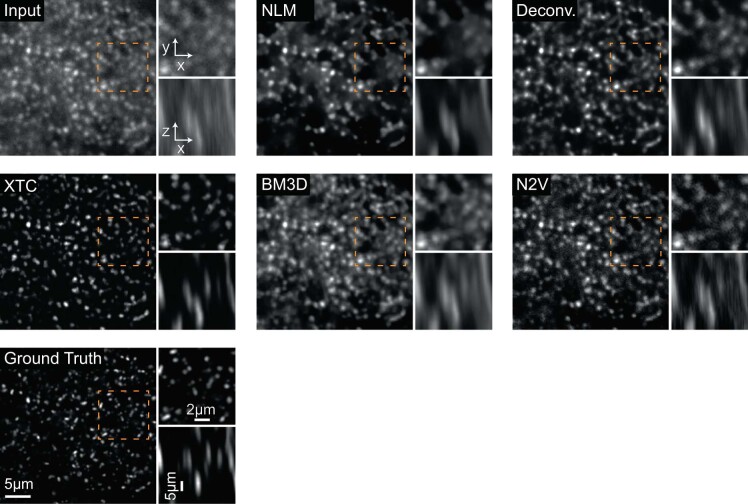
Comparison of XTC to existing algorithms using *slice 2p* data. Comparison of XTC restoration to four algorithms (*NLM*, *Deconvolution*, *BM3D* and *Noise2Void*) using *slice 2p* validation data. Identical ‘Input’ *slice 2p* volume provided to all algorithms. Inset crop from orange boxes shown on right of each comparison, with XY and XZ projections at the top and bottom, respectively. PSNR and NRMSE values for each algorithm output compared to ground truth shown in Fig. 4g, i. Data are representative of larger volume from one SEP-GluA2 mice, examined over one independent experiment.

When we examined the distribution of true-positive, false-negative and false-positive detections between XTC Restored and ground-truth volumes, we observed that the false-positive rate was exceptionally low. Conversely, while the false-negative rate was relatively high, most of these missed detections were either extremely small or dim (Fig. 4e,f). This suggests that image restoration cannot surpass the physical limits of optical elements and thus synapses that are very small or dim (<0.3 µm in diameter) cannot be reliably detected in vivo, even after restoration. Thus, XTC restoration provides high-confidence detection of brighter synapses, with relatively higher AMPAR content. Finally, to further assess the performance of XTC restoration relative to existing alternative methods, we again compared XTC to NLM^28^, Deconvolution^29^, BM3D^35^ and Noise2Void^31,36^. Given the same Slice 2p input, XTC achieved the best image denoising and showed statistically significant improvements in both peak signal-to-noise-ratio (PSNR) and normalized root-mean-squared error (NRMSE) (all comparisons with XTC, PSNR: Slice 2p (*P* = 2.0 × 10^−34^), NLM (*P* = 2.1 × 10^−7^), Deconvolution (*P* = 1.4 × 10^−13^), BM3D (*P* = 10^−22^) and N2V (*P* = 5.7 × 10^−9^); NRMSE: Slice 2p (*P* = 2.0 × 10^−50^), NLM (*P* = 10^−6^), Deconvolution (*P* = 3.9 × 10^−14^), BM3D (*P* = 2.8 × 10^−28^) and N2V (*P* = 2.6 × 10^−8^); one-way analysis of variance with Bonferroni correction for multiple comparisons; Fig. 4g,h and Extended Data Fig. 6).

### Cross-modal image enhancement facilitates synapse tracking

**Fig. 5:**
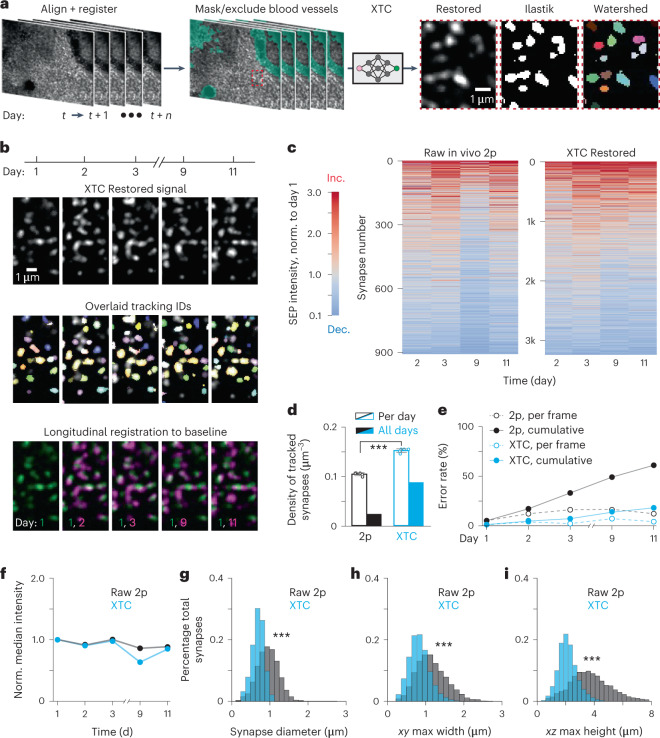
XTC enables tracking of thousands of registered synapses across days during behavior. **a**, Overview of preprocessing pipeline for tracking. Volumes were acquired using in vivo 2p microscopy over 10 d and registered to each other. Blood vessels were masked to facilitate tracking. Imaging volumes were super-resolved with XTC; synapses were detected using ilastik and separated with watershed segmentation. Data are representative of larger volumes from three SEP–GluA2 mice, examined over two independent experiments. **b**, Examples of single-synapse-resolution registration and tracking in vivo. Representative raw signal across ten imaging days (top). Same fields of view with synapse detections overlaid (middle). Identical colors across different days indicate the same tracked synapse. Registered and aligned signals (magenta), relative to day 1 (green) (bottom). Data are representative of larger volumes from three SEP–GluA2 mice, examined over two independent experiments. **c**, Fold change of SEP–GluA2 intensity for individual tracked synapses before and after XTC processing across 11 d of imaging, relative to day 1 baseline. Norm., normalized; Dec., decrease; Inc., increase. **d**, The overall density of synapse detections at each time point and across all time points, was increased after XTC restoration (*P* = 4.0 × 10^−8^, *n* = 4 time points, unpaired two-tailed Student’s *t*-test). Error bars show mean ± s.e.m. **e**, Error rate of tracking for 100 randomly selected synapses, as curated by expert humans, in in vivo 2p and XTC processed volumes. **f**, Median fold change of all synapses at each time point. **g**–**i**, Distribution of diameter in *xy* (*P* = 1.6 × 10^−103^), major axis length in *xy* (*P* = 8.5 × 10^−134^) and major axis length in *xz* (*P* < 0.001) for individual synapses in both Raw 2p and XTC Restored volumes (for all comparisons unpaired two-tailed Student’s *t*-test). ∗∗∗ represents *P* < 0.001.

Accurately tracking synapses over weeks in behaving animals is critical to understand how synaptic changes enable learning and memory. We hypothesized that XTC restoration would facilitate synapse tracking by improving signal-to-noise ratio and reducing ambiguities. To compare synapse tracking before and after XTC, cranial windows were surgically implanted over retrosplenial cortex and animals were imaged over 2 weeks. All time points were then registered volumetrically, followed by a masking step to remove areas obscured by blood vessels. XTC was then applied to each volume, followed by synapse segmentation using a trained ilastik classifier and subsequent synapse tracking using a structured learning algorithm^39,40^ (Figs. 1j and 5a,b).

Synapse tracking was improved when XTC was applied to enhance in vivo 2p volumes. The number of synapses tracked over 10 d was more than threefold higher in XTC Restored volumes (Fig. 5c; 907 versus 3,241 detections) and the overall density of synapses detected at each time point was also increased by 50% (Fig. 5d; 0.106 ± 0.003 mean ± s.e.m. synapses per µm^3^ for in vivo 2p and 0.154 ± 0.004 synapses per µm^3^ for XTC Restored; *P* = 4.0 × 10^−8^; unpaired two-tailed Student’s *t*-test). XTC also increased the density of synapses that could be tracked across all time points to 60% of all synapses in XTC Restored data, compared to 24% in Raw 2p data (Fig. 5d). Moreover, when we manually assessed the rate of tracking errors for 100 randomly selected synapses before and after image enhancement, we also observed a reduced error rate at each individual time point, resulting in a threefold reduction in cumulative errors (Fig. 5e; cumulative error of 18% for XTC and 61% for in vivo 2p on day 10). Additionally, we found that while baseline synapse dynamics were stable across 10 d of imaging in XTC Restored volumes (Fig. 5f), the median fold change for non-restored volumes showed a marked decline on day 9 (Fig. 5c,f), likely due to tracking errors from accumulated tissue shifts that were more difficult to resolve without XTC restoration. We also compared the distribution of sizes of all tracked synapses before and after image restoration and found that the diameter of segmented synapses was closer to their expected physiological size (<1 µm in diameter) in XTC Restored volumes (Fig. 5g–i; 1.0 ± 0.008 µm Raw 2p and 0.77 ± 0.005 µm XTC synapse equivalent diameter; *P* = 1.6 × 10^−103^; 1.2 ± 0.009 µm Raw 2p and 0.77 ± 0.005 µm XTC max *xy* width; *P* = 8.5 × 10^−134^; 1.0 ± 0.008 µm Raw 2p and 0.77 ± 0.005 µm *XTC* max *xz* height; *P* < 0.001 all comparisons mean ± s.e.m.; unpaired two-tailed Student’s *t*-test). Overall, XTC facilitates synapse tracking by substantially denoising images, thereby simplifying the tracking task such that simple, sparsely annotated tracking models can be effectively employed.

### XTC enables tracking of behaviorally induced synapse dynamics

**Fig. 6:**
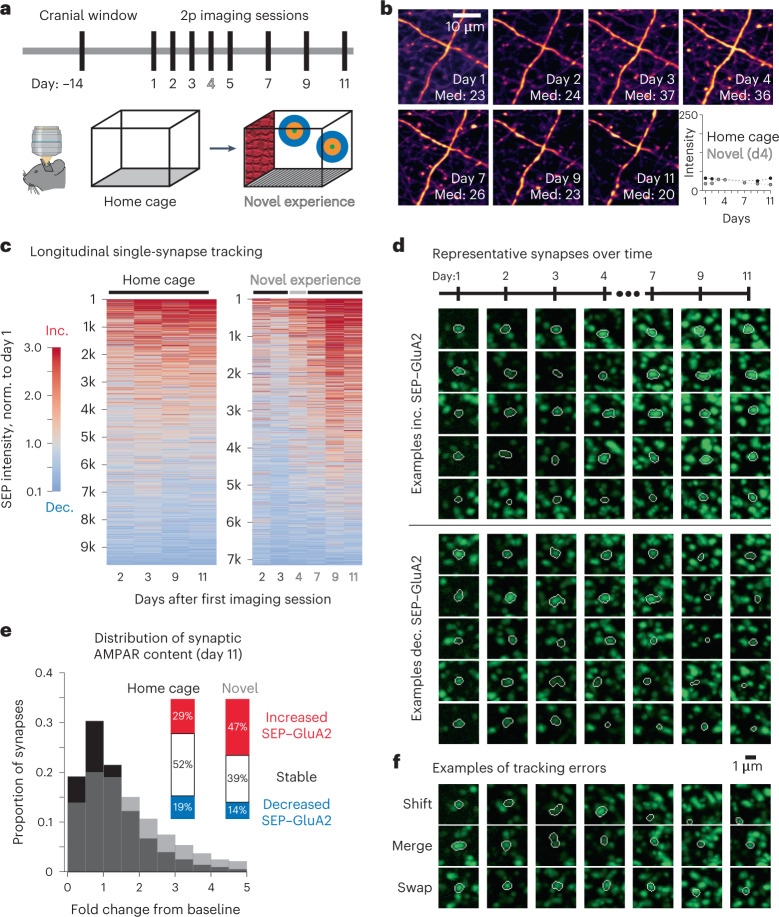
Increased synaptic plasticity in cortex following novel-exposure behavior. **a**, Experimental timeline. Same volumes of retrosplenial cortex were imaged over 11 d. On day 4, animals were exposed to a novel environment for 5 min, consisting of novel textures, smells, sounds and visual cues. **b**, Variations in daily signal intensity were measured and compensated by normalizing SEP signal to red cell fill, excited by the same beam path. Values reported are median intensity values (8 bit range 0–255). Data are representative of larger volumes from two SEP–GluA2 mice, examined over one independent experiment. **c**, Comparison of synapse dynamics in home cage (control) and novel-exposure mice. Fold change relative to day 1 baseline. One mouse is displayed per condition. Data are representative of larger volumes from two SEP–GluA2 mice, examined over one independent experiment. Norm., normalized. **d**, Examples of tracked synapses that displayed increased or decreased strength relative to baseline. Maximum projection of cropped volumes (4 × 4 × 4 µm). All images normalized to red channel signal intensity. Data are representative of ~10,000 tracked synapses from two SEP–GluA2 mice, examined over one independent experiment. **e**, Proportion of synapses that increased, decreased or remained stable after 1 week following novel experience behavior. Inset, quantification of synapses that increased, decreased or remained stable in control and experimental condition. Thresholds of >150% and <50% of day 1 SEP intensity defined increased and decreased SEP–GluA2 expression on day 11, respectively. Data are from one SEP–GluA2 mouse per condition. **f**, Examples of error types in longitudinal synapse tracking. Scale bar refers to all images in **d** and **f**.

To assess whether XTC Restored volumes can be used to track and detect differences in AMPAR content in an established behavioral paradigm for synaptic plasticity, we exposed mice to a novel environment after 3 d of baseline imaging (days 1–3) (Fig. 6a). This behavior (day 4, occurring 2 h before imaging session) consisted of a single, 5-min period of exploration in a chamber containing novel visual, auditory, olfactory and tactile stimuli, after which the mice were transferred back to their home cage. Animals were then imaged on days 5, 7, 9 and 11. To correct for shifts in global signal intensity, SEP intensity was normalized to the red signal of a sparse subset of neurons expressing tdT, as both signals were excited by the same beam and detected with high- and low-pass filters, respectively (910 nm 2p excitation; Fig. 6b).

Animals exposed to a novel environment showed a marked shift in the proportion of synapses with both stronger and weaker synaptic connections (corresponding to increased and decreased synaptic SEP–GluA2 content, respectively) and an overall net increase in AMPAR content, consistent with the net induction of long-term potentiation, which is known to encode spatial learning (Fig. 6c). Representative examples of successfully tracked synapses are shown in relation to the first imaging time point (Fig. 6d). The proportion of synapses that increased in strength, defined as having a sustained fold change > 1.5 on days 9 and 11, was 16.9% in the home cage control compared to 24.3% in novel conditions. Moreover, the proportion of synapses that decreased in strength, defined as having a sustained fold change < 0.5 on days 9 and 11, was also lower after novel exposure (24.0% in home cage and 21.0% in novel conditions; Fig. 6e).

While this XTC-based tracking pipeline was able to detect these biological differences in AMPAR content, we still noted several errors that could be categorized into error types that future algorithms should focus on preventing: ‘shift’ errors, where one association error will continue to propagate on additional time points in regions with poor registration; ‘merge’ errors, which occur at the detection stage and result in blobs of synapses being tracked as a single entity; and ‘swap’ errors, where a tracked synapse incorrectly jumps to a nearby synapse at a later time point (Fig. 6f). These errors could be minimized with more complex detection and tracking models using deep-learning or other machine-learning approaches.

## Discussion

To better understand how behavioral learning is encoded through changes in the strengths of individual synapses, we designed a transgenic mouse line, SEP–GluA2 and developed a computational pipeline to register, super-resolve, identify and longitudinally track individual synapses in vivo. Crucially, our transgenic labeling strategy tags the functional component of excitatory synapses, the AMPARs, which allows researchers to generate detailed maps of synaptic plasticity. Moreover, we showed that by enhancing low-resolution 2p SEP fluorescence to optimal Airyscan-level image quality, it is possible to visualize the AMPAR content of excitatory synapses within a broad cortical region in vivo, providing insight into the spatiotemporal relationship between plasticity and behavioral experience.

At the core of this synapse tracking paradigm is XTC, a cross-modality supervised image-restoration model that substantially improved the axial and lateral resolutions of in vivo 2p images, while retaining the benefits of increased penetrance and imaging speed. XTC restoration also generalized well to unseen data, such as fluorescently labeled neurons and sufficiently reduced the variability between human researchers to enable reliable synapse tracking. Moreover, when compared to a suite of existing denoising algorithms (NLM, Deconvolution, BM3D and Noise2Void), XTC outperformed all other models. This performance highlights the benefits of supervised deep-learning approaches, which in these settings are only possible with a cross-modality training paradigm, for accurate image restoration in vivo.

Beyond synapse tracking, XTC serves as a proof of concept for generating similar supervised restoration algorithms to enhance other in vivo fluorescent signals. Using our slice training paradigm, researchers can rapidly generate trained CNNs to enhance a multitude of in vivo datasets, such as transient signals generated by genetically encoded calcium sensors, fluorescent molecules that are easily photo-bleached or other nanoscale structures that are near the detection limit of in vivo objectives; however, a caveat of this supervised restoration paradigm is that it is still fundamentally limited, as expected, by the resolution of optical elements, as most undetected synapses after XTC were below the noise threshold. Thus, moving beyond the noise threshold requires either higher resolution objectives or other optical/computational components that improve photon capture^41–45^. Additional improvements to synapse tracking can also be realized by developing more complex detection and tracking algorithms. For instance, 3D mask-RCNNs^46^ perform exceptionally well at segmenting individual objects in regions of high density, which can reduce ‘merge’ errors at the detection stage. Moreover, the application of sophisticated registration algorithms, such as LDDMM^47^, could further alleviate the challenge of synapse tracking by aligning all detected synapses automatically.

Overall, the biological and computational tools presented here provide a framework to study synapse plasticity longitudinally at high resolution. Using XTC, future experiments can now readily advance the development of detection and tracking algorithms to interrogate the spatiotemporal relationship between changes in synaptic strength and animal behavior. For instance, detailed spatial analysis can identify the location of subpopulations of synapses that change their synaptic strength in concert across cortical regions. Moreover, combined with Cre-dependent neuronal labeling, researchers can study synaptic plasticity within specific subsets of neurons in the brain. Thus, our computational cross-modality image-restoration paradigm sets the stage for detailed molecular studies of synaptic plasticity underlying behavior, bringing us a step closer to understanding the structural and functional foundations of cognition.

## Methods

### Animal ethics

All surgical and animal procedures were approved by the Johns Hopkins Animal Care and Use Committee. All animal work complied with relevant ethical guidelines. Mice were housed in a climate-controlled room on a 12-h light–dark cycle.

### Mouse genetics

SEP–GluA2 mice were made in collaboration with the Johns Hopkins University School of Medicine Transgenics core, on a C57/BL6 background. Briefly, we used a CRISPR-Cas9-based approach to insert a SEP tag into exon 1 of *Gria2*, localized to the area encoding the GluA2 subunit N terminus. Homozygous transgenic mice are viable, breed well and seem to be physiologically and behaviorally normal. In this transgenic line, all GluA2-containing AMPARs are labeled at endogenous levels, enabling robust visualization of excitatory synapses throughout the entire brain.

### Surgical procedures

Mice were anesthetized (1.5–2% isoflurane) and implanted with a 3 × 3-mm cranial window (Potomac Photonics) over the retrosplenial cortex at 10–16 weeks of age. Windows were sealed and custom-made metal head bars were attached using dental cement (Metabond). In a subset of experiments, an AAV-CaMKII-cre virus (Addgene/Penn Vector) was injected into cortex (viral titer, 5 × 10^8^–1 × 10^9^, 100–150 nl, 0.3 mm deep) of double homozygous SEP–GluA2 × Ai9 reporter mice to sparsely label L2/3 pyramidal neurons with a tdT cell fill. Then, 10 mg kg^−1^ of extended-release Buprenorphine (ZooPharm) was administered before surgery and mice were observed for 3 d following surgery. Mice were allowed to recover for at least 2 weeks before commencing in vivo imaging.

### Mouse behavior

Mice were handled daily for 1 week before behavioral testing. Novel-exposure behavior consisted of a single 5-min exploration session in a novel spatial environment. Mice were individually placed in a 20-cm square chamber with distinct spatial markings and textures on the walls (60-grit sandpaper), a novel smell (70% ethanol), novel cage floor (1-cm-spaced circular bars) and white noise (70 dB). Home-cage control mice were handled daily for 1 week before the start of the experiment but were not exposed to a novel spatial environment. Equal numbers of male and female mice were used for all experiments. Mice used throughout this study were aged 12–20 weeks.

### In vivo and in vitro 2p imaging

in vivo 2p images were acquired from lightly anesthetized mice (1.5% isoflurane) using a custom-built, 2p laser-scanning microscope controlled by ScanImage (Vidrio) and a ×20/1.0 NA water-immersion objective lens (Zeiss). SEP–GluA2 (green) and tdTomato cell fill (red) were both excited at 910 nm with a Ti:sapphire laser (SpectraPhysics, 20 mW power at objective back aperture). Green and red fluorescence signals were acquired simultaneously and separated by a set of dichroic mirrors and filters (ET525/50 m for green channel, ET605/70 m for red channel, Chroma). Image stacks were acquired by resonance scanning at 30-Hz, such that 60 images were captured over 2 s for each *xy* plane. These images were then rigidly aligned (using Stack GPS, https://github.com/ingiehong/StackGPS) to compensate for small movements due to breathing and averaged for each plane. The field of view contained 1,024 × 1,024 × 70 voxels with a lateral *xy* resolution of 0.096 μm per px and an axial resolution of 1 μm per px. Live, 300-μm thick acute slices of SEP–GluA2 brains were imaged using the same optical setup, except that the tissue was held in place with a platinum/nylon harp, as described for 1p imaging. Slices were maintained in HEPES-buffered artificial cerebrospinal fluid (ACSF), consisting of 140 mM NaCl, 5 mM KCl, 10 mM glucose, 10 mM HEPES, 2 mM CaCl_2_ and 1 mM MgCl_2_, with pH adjusted to 7.40.

### 1p confocal and Airyscan imaging

Paired high-resolution Airyscan and low-resolution confocal training volumes were generated using a Zeiss 880 microscope and a ×63/1.0 NA water objective lens (Zeiss) in live-slice preparations. Homozygous SEP–GluA2 mice were transcardially perfused with ice-cold, oxygenated ACSF, the brain was removed and 300-μm thick acute coronal slices of dorsal cortex were made. Slices were incubated in 32 °C oxygenated ACSF for 45 min and then maintained in oxygenated ACSF at room temperature. During imaging, slices were held in place with either a platinum/nylon harp or rapid annealing UV-activated optical glue. Data collection used Zen Black software (Zeiss).

SEP–GluA2 and tdT cell fill were excited at 488 nm and 546 nm, respectively. Optimal high-resolution images were acquired using calibrated Airyscan detectors that achieved a lateral resolution of 0.063 μm per px and an axial resolution of 0.33 μm per px. Immediately after high-resolution volumes were imaged, paired suboptimal images were acquired to reduce registration errors. The image quality of suboptimal images was curated to replicate the image quality of in vivo 2p datasets by opening the confocal pinhole four-times higher than the ideal AU, increasing the laser gain to near maximal levels and reducing laser power. Overall, we collected 24 paired high–low-resolution training volumes with a 550 × 550 × 20 voxel field of view from eight tissue slices containing multiple cortical regions. Validation and all in vivo data were generated from different animals (*n* = 5 mice). In addition to live-slice imaging, we also applied the Zeiss 880 microscope in Airyscan Fast mode^33^, with a ×20/1.0 NA water-immersion objective lens (Zeiss), to attempt to detect synapses in vivo.

### Neural network architecture and optimization

Having collected pairs of aligned high- and low-resolution images, $\{ x_{\mathrm{l}}^i,x_{\mathrm{h}}^i\} _{{\mathrm{i}} = 1}^N$, we employed a supervised learning approach to find a map from input images $x_{\mathrm l} \in R^{n_\mathrm{l}}$ to outputs $x_{\mathrm h} \in R^{n_{\mathrm{h}}}$, where $n_{\mathrm l} < n_{\mathrm{h}}$. In particular, we parametrize this function $f_\theta :R^{n_{\mathrm l}} \to R^{n_{\mathrm h}}$ with a CNN with parameters *θ*. We employ a CNN architecture similar to those proposed by Weigert et al. with a modified U-Net architecture^1,4^. Following previous work^48^, the input is first bicubic-interpolated to match the target dimension before applying a function parameterized by the CNN. At deployment, in vivo volumes follow an analogous pipeline whereby they are first interpolated to match the axial and lateral resolutions of the training data before restoration. Following an empirical risk minimization approach, we minimize a suitable loss function over the training samples according to:

$$f_{\hat \theta } = {\mathrm{arg}}\mathop {\mathrm {{min}}}\limits_\theta \frac{1}{N}\mathop {\sum}\limits_{i = 1}^N l \left( {f_\theta \left( {x_{{\mathrm{l}}}^{i}} \right),x_{\mathrm{h}}^{i}} \right)$$

The loss penalizes deviations by the reconstructed images, $f_\theta \left( {x_{\mathrm l}^I} \right)$, from the high-quality samples, $x_{\mathrm h}^i$. For simplicity, we chose to optimize the average of mean absolute error, namely $l\left( {f_\theta \left( {x_{\mathrm l}^i} \right),x_{\mathrm h}^i} \right) = \lambda f_\theta \left( {x_{\mathrm l}^i} \right) - x_{\mathrm h1}^i$. Combining this with other losses, such as the multiscale structural similarity index (MS-SSIM)^49^, is certainly possible and might provide further improvements. Overall, we trained a CNN with paired high-resolution Slice Airy and low-resolution Slice 1p data for 1,000 epochs with batch size of eight on an NVIDIA Tesla P100-PCIE GPU using an Adam optimizer^50^ and a learning rate of 4 × 10^−4^.

### Validation comparisons

The ideal validation experiment would require imaging the same field of view first in vivo, using our 2p setup and then again ex vivo in slice, with Airyscan detectors. This setup would allow us to directly compare synapses detected in XTC Restored in vivo images to synapses in ground-truth Slice Airy volumes. Unfortunately, we found that it was extremely difficult to find the exact same field of view ex vivo post-perfusion, even with the addition of structural anchors to help with registration, such as sparse neuronal labels. Moreover, we found that it was entirely impossible to preserve the tissue throughout dissection such that the position of synapses remained stable enough for registration. Thus, we chose to perform our validation in live-slice tissue directly, where we could try to faithfully replicate the quality of images acquired in vivo by using the same 2p microscope, resonance scanner, objective and laser power to acquire our low-resolution Slice 2p images (Fig. 1h,i). Moreover, tissue slices were imaged immediately after dissection to preserve endogenous tissue quality. The high-resolution images (Slice Airy) paired to these low-resolution Slice 2p volumes were acquired on a separate Zeiss 880 microscope equipped with Airyscan detectors (Fig. 1g). The high- and low-resolution validation pairs were then registered together using a combination of FIJI’s correct 3D drift package^51^ and SimpleElastix affine transformations^52^ to remove tissue movements that occurred when transferring between microscopes.

Finally, we compared pairwise detected synapses, segmented using ilastik voxel classifiers, between high-resolution Slice Airy volumes and both Slice 2p and XTC Restored volumes. For these comparisons, we defined ‘true-positive’ detections as synapses sharing at least one voxel across the pairwise compared volumes. As this threshold was very lenient, we also included several validation metrics to assess pairwise structural and intensity similarities to validate the extent to which XTC processing improved the size and shape of synaptic detections.

### Optimization of alternative denoising algorithms for comparison

Four additional image-denoising algorithms were used to evaluate the performance of XTC. These four algorithms were applied to the exact same in vivo 2p and Slice 2p data provided to XTC, as indicated in Fig. 3a and Extended Data Fig. 6 and each algorithm was optimized for analysis in the following ways:

1. NLM^28^: we implemented the version of NLM packaged in Python’s scikit-image library using the ‘slow mode’ of operation. The performance of the algorithm was adjusted by optimizing the sigma value (noise standard deviation) between a range of 10 to 30. We then applied the algorithm slice by slice, as the algorithm was not optimized for large volumetric data.
2. Deconvolution: we used the DeconvolutionLab2^29^ package (available in FIJI) to perform Lucy–Richardson Deconvolution^53,54^. To estimate the point-spread function, we used DeconvolutionLab2’s point-spread function generator package and selected the Richards and Wolf 3D Optical Model. We provided the refractive index, wavelength, NA and voxel size from our optical setup. To optimize the performance of Deconvolution, we altered the number of iterations performed by the Lucy–Richardson algorithm between the range of 2 to 20. We selected ten iterations as the optimal performance.
3. BM3D^35^: we used the MATLAB implementation of BM3D and started the algorithm in ‘all levels’ mode to enable multilevel denoising. For optimization, we adjusted the sigma value between value 5 and 50.
4. Noise2Void^31,36^: a state-of-the-art unsupervised image-denoising algorithm, which is conveniently offered by ZeroCostDL4Mic^36^. Using this platform, we provided one training volume of size (35 × 35 × 70 µm) and trained for 24 h (100 iterations) on a GPU 2080 RTX Ti with all other default settings. The performance was then established by applying the ‘best’ model, as detected by default in Noise2Void.

### Registration and data processing for synapse detection and longitudinal tracking

Several preprocessing steps were applied to facilitate longitudinal synapse tracking (Fig. 1j). First, each image at a given time point *t* was volumetrically registered to the subsequent time point *t* + *1* using affine transformations in SimpleElastix. While volumetric registration accounted for global tissue shifts, we found that local misalignments persisted after volumetric registration. Thus, we included an additional registration step that registered each *xy* slice on time point *t* with each corresponding slice on time point *t* + *1*. The final preprocessing step was to detect blood vessels and exclude them from our analysis, as synapses located adjacent to blood vessels could easily become obscured and appear as an eliminated synapse. To perform blood vessel masking, volumes were binarized, followed by inversion, image opening and dilation to extract a smooth binary mask that excluded dim dark regions. No other preprocessing steps were applied to the raw data and the registered volumes were then processed with XTC.

Synapse detection and tracking algorithms were trained in ilastik, a platform that enables researchers to rapidly build machine-learning models using sparse annotations. For synapse detection, random forest classifiers were trained, each using 30 human segmented synapses, for all imaging modalities (Slice 2p, Slice Airy, in vivo 2p and XTC Restored), respectively. For synapse tracking, we trained two models using structured sparse learning in ilastik, each with 100 human annotated tracks, for in vivo 2p data both before and after XTC restoration. To ensure that the intensity of the SEP signal compared across tracking experiments is not altered by XTC processing, we also overlaid the XTC segmentations onto the raw in vivo 2p data to extract intensity values directly from the raw data in longitudinal imaging experiments.

### Statistical analysis

All statistical analysis was performed using Python statsmodels and scipy libraries. *n* represents the number of animals used in each experiment, unless otherwise noted. Data are reported as mean ± s.e.m. or median ± s.e.m. as indicated and *P* < 0.05 was considered statistically significant. Level of significance is marked on figures as ^∗^*P* < 0.05; ^∗∗^*P* < 0.01; ^∗∗∗^*P* < 0.001.

### Reporting summary

Further information on research design is available in the Nature Portfolio Reporting Summary linked to this article.

## Online content

Any methods, additional references, Nature Portfolio reporting summaries, source data, extended data, supplementary information, acknowledgements, peer review information; details of author contributions and competing interests; and statements of data and code availability are available at 10.1038/s41592-023-01871-6.

## Supplementary information

Reporting summary

Supplementary Video 1 Volumetric SEP–GluA2 fluorescence in vivo before and after XTC restoration. Volumetric maximum intensity projection generated using Imaris software. The video alternates between low-resolution 2p and XTC Restored volumes as indicated in the top right corner. Scale bar adaptively shown in bottom left. Total volume 100 × 100 × 70 µm.

Supplementary Video 2 Slice-by-slice comparison of SEP–GluA2 volume before and after XTC. Identical volumes of SEP–GluA2 tissue imaged in vivo, before (left) and after (right) applying XTC. Depth 70 μm, 1-μm steps, starting from the surface and moving deeper into the brain.

Supplementary Video 3 Comparison of XTC and existing denoising algorithms in vivo. Identical input volume (top left) is provided to all algorithms for denoising. Total volume shown 30 × 30 × 70 µm. Outputs of each algorithm as shown slice by slice.

## Data Availability

The datasets generated or analyzed during the current study are available from the following OSF repository: https://osf.io/qdpty/?view_only=c250e8676a434899964cb4e5de676e0d.

## Code availability

Packaged software code is available at github.com/yxu233/Xu_and_Graves_XTC_syn_tracking under the MIT license, along with instructions for use and demo data across a small volume. The algorithm is prepared to work with minimum Python v.3.6. The code is also archived in a Code Ocean capsule at 10.24433/CO.8379773.v1^55^.
